# Occupational Stress Risk and Its Impact on Job Performance Among Bahraini Healthcare Workers: A Cross-Sectional Study

**DOI:** 10.7759/cureus.76413

**Published:** 2024-12-26

**Authors:** Amal Alafoo, Zahra M Mohamed, Adel M Yusuf, Hajar Mubarak, Shaikha Alaradi, Anisa Al-Malali

**Affiliations:** 1 Psychiatry, Government Hospitals (Psychiatric Hospital and Salmaniya Medical Complex), Manama, BHR

**Keywords:** bahrain, burnout, healthcare workers, job performance, occupational stress, quality of care, workplace factors

## Abstract

Introduction

Occupational stress has become increasingly prevalent in the health sector in recent years. This stress poses significant risks, affecting not only the well-being of healthcare workers but also the quality of care patients receive. Therefore, this study aims to assess the prevalence of occupational stress among health workers, identify its roots, and examine its effects on productivity.

Methods and materials

Data were collected through an electronic survey process from several healthcare professionals employed at two governmental hospitals in the Kingdom of Bahrain. The participants were selected through a random sampling approach, targeting healthcare professionals who met specific inclusion criteria. These criteria required the participants to have at least one year of professional experience, work a minimum of 20 hours per week, and be involved in direct patient care. The research team invited eligible participants via email, ensuring the representation of diverse roles, including doctors, nurses, and allied health professionals. Excluded roles included administrative staff, pharmacists, and laboratory technicians, who have limited patient interaction. The survey, completed voluntarily and anonymously, included 46 questions that focused on work demographics, the Perceived Stress Scale (PSS), and the Individual Work Performance Questionnaire (IWPQ). Occupational stress and job performance were analyzed as dependent variables, with statistical analysis conducted using the Statistical Package for Social Sciences version 23 (IBM Corp., Armonk, NY).

Results

Most of the health workers were found to experience moderate to high levels of occupational stress, with a prevalence rate of 90.2%. Despite this, the majority (63.1%) satisfactorily performed their job duties. Moreover, a significant negative correlation was observed between job performance and occupational stress (r = -0.965).

Conclusion

This research reveals that occupational stress among Bahraini health workers is high and leads to reduced job performance.

## Introduction

Occupational stress poses a serious risk to health workers, significantly affecting their job performance and overall well-being [1]. Health workers, including nurses, doctors, and other healthcare professionals, face high levels of occupational stress owing to the demanding nature of their work environment [1].

Stress is a complex condition that results from the appraisal of the demands of an individual and their resources and ability to meet those demands [1]. It manifests as a mental, physical, and emotional strain, often triggered by different aspects such as workload, work-life balance, and workplace resources [2]. Non-organizational factors, including trauma, social support, and personal well-being, also contribute to stress [3]. Increasingly, healthcare managers recognize that stress is a major occupational hazard, influencing job satisfaction, performance, the health of a patient, and the overall quality of the services provided [1]. High stress levels impair cognitive function, decision-making capacity, and emotional regulation, resulting in mistakes, close calls, and poor performance [4]. This indicates a greater level of risk in critical settings such as hospitals, where errors can lead to severe consequences.

Burnout, classified as an "occupational phenomenon" in the 11th Revision of the International Classification of Diseases [5], is a significant issue in the workplace. Girma et al. report that this condition negatively affects the well-being of health workers, their job performance, and the quality of care they deliver to patients [6].

The causes of occupational stress among healthcare workers are diverse and closely related to factors such as workload, long hours, emotional demands, resource availability, policies and procedures, interpersonal conflicts, job ambiguity, and personal issues such as family or financial pressure [7]. These stressors could lead to a range of adverse outcomes, including burnout, depressive and anxiety symptoms, poor physical health, high turnover and absenteeism rates, lower job satisfaction, and reduced care and attention for patients [7]. Additionally, chronic occupational stress is associated with higher morbidity and mortality rates among healthcare workers, highlighting the urgent need for intervention [8].

Job performance refers to the degree to which an employee can meet the required standards of proficiency in performing organizational activities [9]. Health workers play a crucial role in the healthcare team, dedicated to ensuring the delivery of high-quality care to patients, clients, or communities [10]. The job performance of health workers is a complex concept encompassing various dimensions. It involves their ability to provide quality services in a standard and competently professional manner [11]. Key performance indicators (KPIs) for assessing health worker performance include the satisfaction of the patients and the quality of services provided [12].

This study aims to outline the key facets of job performance, including communication, technical competence, problem-solving skills, time management, teamwork, adherence to standards, emotional intelligence, and learning acquisition. Hence, communication is vital for conveying information clearly to patients and other healthcare workers [12]. Technical competence refers to performing job tasks accurately and safely [13]. Core cognitive skills include problem-solving, essential for making the right decisions under pressure, and time management, which supports the effective organization of activities and projects [14]. Collaboration with other healthcare providers is critical to delivering comprehensive care [14]. Adherence to guidelines and procedures ensures consistency and optimal patient outcomes [14]. In contrast, emotional intelligence allows healthcare workers to understand and manage emotions, especially when interacting with patients [15]. Further education is necessary to stay updated on the best practices and modern technologies for effective interventions [16].

The job performance of healthcare workers is crucial to the quality of patient care (QPC) in Bahrain [17]. Occupational stress is a significant concern for healthcare workers in this region [18]. High stress levels among Bahraini healthcare workers are attributed to factors such as heavy workload, long hours/shift work, insufficient support from colleagues/supervisors, the lack of essential resources and equipment, high patient-to-nurse ratios, poor communication/interpersonal conflicts, and fear of making mistakes or facing legal repercussions [19]. Moreover, other factors, such as role ambiguity and conflicts, further contribute to their stress [19].

Occupational stress may also result in increased errors, endangering the safety of the patient and compromising service quality [20]. Stress also lowers morale, negatively affecting the overall team performance and the organizational culture of the hospital [20]. Furthermore, occupational stress contributes to mental health issues, including depression and anxiety, as well as physical health challenges, including cardiovascular disease, gastrointestinal complaints, and musculoskeletal conditions [18,21].

Significance of the study

Healthcare professionals in Bahrain face demanding conditions, including long working hours, emotionally stressful responsibilities, and critical life-threatening decisions. Occupational stress has serious implications, affecting the mental and physical health of these workers and ultimately influencing their job performance. This study explores how occupational stress affects Bahraini healthcare providers, particularly in relation to their work aspects, personal well-being, and the quality of patient care (QPC). The findings reveal high levels of occupational stress among Bahraini healthcare practitioners, emphasizing the need to understand its contributing factors and their impact on job performance. These insights can guide decision-makers in developing strategies to reduce workload, promote work-life balance, and strengthen support systems for healthcare workers. By identifying sources of occupational stress, healthcare organizations can implement evidence-based interventions to enhance employee well-being and job performance. Additionally, examining the relationship between organizational factors and occupational stress is crucial for designing effective approaches to mitigate stress-related effects on job performance and ultimately improve QPC.

Aim of the study

This study aims to assess the extent of occupational stress among Bahraini healthcare workers, identify its causes, and evaluate its effect on job performance. Overall, the research aims to determine the prevalence of occupational stress among Bahraini healthcare providers, identify personal characteristics and workplace factors that contribute to high levels of professional burnout, and examine the relationship between job strain and productivity in this population.

Questions of the study

What is the prevalence of occupational stress among Bahraini healthcare workers? What demographic and occupational factors predict occupational stress in this group? Does a significant relationship exist between occupational stress and job performance among Bahraini healthcare workers? Are there differences in occupational stress and job performance between various types of healthcare workers (e.g., nurses, doctors, and support staff)? Are there any differences in occupational stress and job performance between different hospital settings?

Research questions

What is the prevalence rate of occupational stress among healthcare professionals in Bahrain? Which personal or professional demographics and workplace factors predict stress among allied healthcare professionals (AHPs)? Is there a correlation between burnout levels and productivity among AHPs? Do AHPs experience chronic fatigue differently compared to other healthcare workers?

## Materials and methods

Study design

A cross-sectional descriptive design was utilized to investigate the effect of occupational stress on job performance.

Setting

This study was conducted in two government healthcare facilities in Bahrain: Salmaniya Medical Complex and the Psychiatric Hospital.

Sample

The participants were healthcare professionals employed at Salmaniya Medical Complex and the Psychiatric Hospital in Bahrain, selected based on specific inclusion and exclusion criteria. Inclusion criteria required the participants to have no less than one year of professional experience and work at least 21 hours per week across all shifts. Eligible roles included doctors (physicians), registered nurses, medical assistants, respiratory therapists, psychologists, occupational therapists, physical therapists, speech-language pathologists, mental health professionals, dietitians/nutritionists, and allied health professionals directly involved in patient care. Exclusion criteria excluded roles with limited or indirect patient interaction, such as pharmacists, laboratory technicians, phlebotomists, health unit coordinators, administrative staff, service workers, and transportation staff. Participation in the study was voluntary, with no financial or material incentives provided.

Procedure and data collection

A Google Form was created for data collection in this study. Assistant directors formed the sample collaboratively by messaging potential participants and inviting them to take part in the survey. This invitation included an outline of the objectives of the study, details on data collection procedures, and assurances of confidentiality, as all responses were anonymous. The participants were provided with a link to the Google Form and instructed to confirm receipt of the invitation by email before accessing and completing the survey. The submission deadline was set at two weeks from the date of invitation. All electronic data were then securely stored on a password-protected server accessible only to the researcher. Permission to use assessment tools was obtained directly from the authors by the researcher. Each participant took approximately 20-30 minutes to complete the survey, with data collection running from July to August 2024.

Instruments

To achieve the goals of the study, a modified self-report questionnaire was used to collect data and identify study variables. The questionnaire was divided into the following sections: occupational demographics, the Perceived Stress Scale (PSS), and the Individual Work Performance Questionnaire (IWPQ) (Appendices).

The occupational demographics section comprised demographic characteristics (age, sex, professional status, marital status, workplace, education, and years of experience) and career-related issues with 10 questions. These questions included workload and intensity, control and autonomy, light work, community support, emotional and physical needs, expectations and feedback, work-life balance, organizational culture, and supervisor support (Appendices).

The PSS is a broadly utilized, standardized measure of perceived stress that offers a comprehensive understanding of an experience with stress, including tangible and emotional perspectives [22]. This self-report instrument evaluates the perceived stress levels of an individual over the past month. The PSS comprises 10 items rated on a five-point Likert scale, ranging from "never" to "very often." The scale includes two subscales: the five-item "time weight and overpowering assignments" and the five-item "need of control and vulnerability" subscales [23]. The time weight and overpowering assignments subscale (items 1, 3, 5, 7, and 9) assesses the ability of an individual to manage daily demands, including task management, deadlines, and responsibilities. In contrast, the need of control and vulnerability subscale (items 2, 4, 6, 8, and 10) focuses on the emotional and psychological aspects of stress, measuring the sense of control over life events and circumstances. The PSS total score ranged from 0 to 40, with higher scores indicating higher perceived stress. Scores from 0-13, 14-26, and 27-40 indicate low stress, moderate push, and high perceived stretch, respectively. The PSS has demonstrated consistency and validity, with studies reporting strong internal consistency (Cronbach's alpha = 0.84-0.92) and test-retest unwavering quality (r = 0.74-0.85) (Appendices).

The IWPQ is an 18-item standardized instrument designed to evaluate the job performance of an individual across the following main subscales: task performance (TP; five items), contextual performance (CP; eight items), and counterproductive work behavior (CWB; five items) [24]. The IWPQ helps in understanding the work performance of an individual, identifying areas of strength and weakness, and creating targeted mediations to improve work performance and overall organizational effectiveness [25]. The TP subscale measures the capacity of an individual to complete tasks productively and viably. The five items assess perspectives such as planning, prioritizing, and executing work tasks. The CP subscale focuses on behaviors that extend beyond essential work duties, reflecting a commitment to the organization and coworkers. The eight items evaluate behaviors such as taking initiative, seeking new challenges, and sharing knowledge and skills with others. The CWB subscale measures behaviors that are harmful to the organization or colleagues, including complaining, gossiping, or sabotaging others. The five items evaluate behaviors that may negatively affect the work environment or interpersonal relationships. For performance evaluation, scores range as follows: Unsatisfactory performance falls between 13 and 38 points, whereas satisfactory performance falls between 39 and 65 points. The IWPQ has demonstrated dependability and validity, with high internal consistency (Cronbach's alpha ranging from 0.73 to 0.94) and moderate to high test-retest reliability (correlations from 0.63 to 0.85 across subscales). Moreover, it shows strong convergent validity, with relationships between 0.45 and 0.80 with other work performance measures (Appendices) [24].

Pilot study

A focus group discussion was conducted with 30 health workers to assess the feasibility, usability, and potential challenges of the study tool. The pilot study also allowed for adjustments to the questionnaire, with permission to use it granted if any necessary corrections were identified. The internal consistency of the tool was then established by calculating Cronbach's alpha, which resulted in a score of 0.83, indicating strong consistency and accuracy.

Method of data collection

Ethical Approval

Prior to the study, research permission was obtained from the Research Committee of the Government Hospitals on 7 May 2024, with serial number 56-070524. The participants received a detailed email outlining the purpose of the study, their rights, the survey process, and the procedures for survey termination. They were informed that all the responses were completely anonymous and their identities would be kept confidential. Contact information was provided for further questions. The participants were assured of their right to choose whether to participate, with the option to decline without consequences. Furthermore, the results of the study were emphasized to be reported with full respect for the confidentiality of the participants.

Data Analysis

Data were carefully reviewed for completeness and accuracy before coding and entry. Descriptive statistics, including percentages, means, and standard deviations, were used to summarize the sample characteristics and survey scores. Independent sample t-tests, followed by post hoc comparisons using the least significant difference method and the one-way analysis of variance, were conducted to identify significant differences in mean scores, with significance at p < 0.05.

## Results

The questionnaire was distributed to over 700 healthcare workers across two hospitals, with 409 of 715 respondents completing the survey, resulting in a response rate of >58%. Incomplete samples were dismissed from the analysis to maintain the integrity and consistency of the dataset. The participants were primarily from Salmaniya Medical Complex and the Psychiatry Hospital. Regarding sex distribution, 11 more women participated in the study, with men accounting for 45% of the respondents. The professional experience of the participants was mostly represented by doctors (49.1%), nurses (43.3%), and other healthcare roles (7.6%). Marital status data showed that most participants were married (83.6%), while 15.2% were single. Education levels varied, with 71.1% holding a bachelor's degree; however, 2.7% and 2.62% had a diploma and completed post-graduate studies, respectively. The age distribution showed that the largest group was aged 30-39 years (52.3%), followed by those aged 40-45 years (34.0%), and then the youngest group aged 20-29 years (13.7%). The mean age was 35.17 years. Finally, regarding the years of experience, 69.7% of the participants had >5 years of experience, with 7% having >11 years of experience in their field, averaging 14.74 years of experience overall. In summary, the findings suggest that the participants are primarily women, college-educated, experienced, and married (Table 1).

**Table 1:** Sociodemographic features of the participants (N: 409) M, mean; SD, standard deviation

		N	%
Hospital	Salmaniya Medical Complex	223	54.5
	Psychiatric Hospital	186	45.5
Sex	Male	184	45.0
	Female	225	55.0
Professional	Doctor	201	49.1
	Nurse	177	43.3
	Others	31	7.6
Marital status	Married	342	83.6
	Single	62	15.2
	Others	5	1.2
Education level	Diploma	11	2.7
	Bachelor's	291	71.1
	Post-graduate	107	26.2
Age	20-29	56	13.7
	30-39	214	52.3
	40-45	139	34.0
		M: 35.17 (SD: 5.765)	
Years of experience	1-5	56	13.7
	6-10	68	16.6
	11	285	69.7
		M: 14.74 (SD: 6.974)	

The research data (Table 2) highlight the factors present in the workplace influencing health workers. Most (63.7%) reported feeling a heavy workload and high working intensity, while 76.6% indicated that they planned their working hours and breaks. The participants generally understood their roles and received adequate support, as reflected in high rates of role clarity (88.0%) and social support (92.0%). However, only 6.8% of the respondents felt emotionally exhausted, and nearly half (49.0%) reported physical overwork. Furthermore, an overwhelming 97.1% of the respondents felt that their achievements were recognized and valued. Regarding work-life balance, 76.1% found it unsatisfactory. However, positive results were observed: 87.6% expressed confidence in the organizational culture, and 87.3% felt supported by their supervisors.

**Table 2:** Workplace factors (N: 409)

The factors	Questionnaire items	Yes		No	
		N	%	N	%
Workload and workload intensity	Do you feel overwhelmed by your workload most days?	261	63.7	148	36.1
Control and autonomy	Do you have control over your schedule and breaks?	314	76.6	95	23.2
Role clarity	Is your role clearly defined and understood by colleagues and supervisors?	361	88.0	48	11.7
Social support	Do you feel supported by your colleagues and supervisor during challenging times?	377	92.0	32	7.8
Emotional demands	Do you feel emotionally drained after interacting with difficult patients or families?	28	6.8	381	92.9
Physical demands	Do you experience physical discomfort or pain due to the demands of your work?	201	49.0	208	50.7
Expectations and feedback	Do you feel like your achievements are recognized and valued by colleagues and supervisors?	398	97.1	11	2.7
Work-life balance	Do you feel like you are able to maintain a healthy balance between work and personal life?	97	23.7	312	76.1
Organizational culture	Are employees encouraged to speak up and share concerns or suggestions for improvement?	359	87.6	50	12.2
Supervisor support	Do you feel supported by your supervisor in terms of resources, guidance, and mentorship?	358	87.3	51	12.4

The majority of the participants (76.3% or 312 individuals) reported experiencing moderate stress, suggesting that while their stress levels are moderately high, they consider it manageable. Furthermore, 57 participants (13.9%) reported high stress, while 40 participants (9.8%) reported low stress. The mean PSS score was 19.80 (6.050), indicating a moderate to high level of stress, with the standard deviation reflecting variability in stress perception among the participants (Table 3).

**Table 3:** Occupational stress distribution (N: 409) PSS, Perceived Stress Scale; SD, standard deviation

PSS scores	N	%
Low	40	9.8
Moderate	312	76.3
High	57	13.9
Mean (SD)	19.80 (6.050)	

Among the sample, 151 participants (36.9%) had unsatisfactory job performance, while 258 participants (63.1%) showed satisfactory performance. The mean score on the IWPQ was 42 out of a possible 60, with a standard deviation of 8.593, indicating moderate variability in job performance among employees (Table 4).

**Table 4:** Job performance (N: 409) IWPQ, Individual Work Performance Questionnaire; SD, standard deviation

IWPQ scores	N	%
Unsatisfactory performance	151	36.9
Satisfactory performance	258	63.1
Mean (SD)	42.93 (8.593)	

A significant negative correlation was found between job performance and occupational stress, with an r value of -0.965 and a significance level of 0.01. This strong negative relationship suggests that as stress levels increase, job performance tends to decline considerably. Moreover, the obtained results showed that this relationship was highly significant at p < 0.000; together, these findings indicate that the association between the two variables is unlikely attributed to chance (Table 5).

**Table 5:** Correlations between job performance and occupational stress *Correlation is significant at the 0.01 level (two-tailed) PSS, Perceived Stress Scale; IWPQ, Individual Work Performance Questionnaire

		PSS scores	IWPQ scores
PSS scores	Pearson correlation	1	-0.965*
	Significance (two-tailed)	-	0.000
	N	409	409
IWPQ scores	Pearson correlation	-0.965*	1
	Significance (two-tailed)	0.000	-
	N	409	409

Job performance levels differed significantly based on relationship status (p = 0.002), workload (p = 0.040), role definition (p = 0.032), expectations and feedback (p = 0.010), organizational culture (p = 0.000), and supervisory support (p = 0.000). Stress levels, meanwhile, were significantly influenced by education level (0.013), years of experience (0.022), workload intensity (0.018), emotional demands (0.037), expectations and feedback (0.029), organizational culture (0.002), and supervisor support (0.001) (Table 6).

**Table 6:** Effect of demographics and workplace variables

Variables	Occupational stress		Job performance	
	F	P value	F	P value
Hospital	3.612	0.058	2.945	0.087
Sex	0.231	0.631	0.000	0.989
Professional	2.797	0.062	0.974	0.378
Marital status	0.543	0.581	6.337	0.002
Education level	4.364	0.013	0.670	0.512
Age	2.499	0.083	2.468	0.086
Years of experience	3.853	0.022	1.432	0.240
Workload and workload intensity	5.643	0.018	4.249	0.040
Control and autonomy	0.906	0.342	3.855	0.050
Role clarity	1.599	0.207	4.608	0.032
Social support	0.400	0.527	1.150	0.284
Emotional demands	4.367	0.037	0.293	0.588
Physical demands	0.005	0.943	1.137	0.287
Expectations and feedback	4.775	0.029	6.692	0.010
Work-life balance	1.451	0.229	2.697	0.101
Organizational culture	10.023	0.002	13.200	0.000
Supervisor support	12.054	0.001	13.849	0.000

The participants with the highest percentage of satisfactory performance were estimated at 50.1%. Workload intensity also affected performance: 37.9% of employees who reported a heavy workload showed satisfactory performance, compared to that of the 25.2% who reported a manageable workload. Additionally, 54% of the participants who reported role clarity attained satisfactory results, compared to that of 9% who lacked role clarity. Expectations and feedback also play a crucial role in influencing performance, as 60% agreed with the statement. Furthermore, 4% of those who received positive feedback performed satisfactorily, in contrast to only 2% of those given negative feedback. Supervisory support and organizational culture showed similar effects; 52.6% and 52.3% of the participants reported satisfactory performance when these factors were present (Table 7).

**Table 7:** Variables affecting job performance

Variables		Unsatisfactory performance		Satisfactory performance	
		N	%	N	%
Marital status	Married	137	33.5%	205	50.1%
	Single	11	2.7%	51	12.5%
	Others	3	0.7%	2	0.5%
Workload and workload intensity	Yes	106	25.9%	155	37.9%
	No	45	11.0%	103	25.2%
Role clarity	Yes	140	34.2%	221	54.0%
	No	11	2.7%	37	9.0%
Expectations and feedback	Yes	151	36.9%	247	60.4%
	No	0	0.0%	11	2.7%
Organizational culture	Yes	144	35.2%	215	52.6%
	No	7	1.7%	43	10.5%
Supervisor support	Yes	144	35.2%	214	52.3%
	No	7	1.7%	44	10.8%

The education level played a significant role, with 52.1% of those holding a bachelor's degree reporting moderate stress. The years of experience also showed a clear pattern, especially among the participants with 11+ years of experience, in which 50.6% experienced moderate stress. Workload intensity was another critical factor; although many respondents experienced workload-related stress, the majority classified it as moderate (47.7%). Emotional demands had a lesser influence on overall stress levels, as most respondents reported low emotional demands and moderate stress levels. The expectations and feedback, along with organizational culture, were positively associated with moderate stress levels, as most respondents expressed positive attitudes toward expectations and feedback while experiencing moderate stress. Supervisor support emerged as another decisive factor; the participants who perceived strong supervisor support reported high stress levels, aligning with their self-reported moderate stress levels (Table 8).

**Table 8:** Variables affecting the stress level

Variables		Low stress level		Moderate stress level		High stress level	
		N	%	N	%	N	%
Education level	Diploma	0	0.0%	6	1.5%	5	1.2%
	Bachelor's	36	8.8%	213	52.1%	42	10.3%
	Post-graduate	4	1.0%	93	22.7%	10	2.4%
Years of experience	1-5	0	0.0%	50	12.2%	6	1.5%
	6-10	10	2.4%	55	13.4%	3	0.7%
	11	30	7.3%	207	50.6%	48	11.7%
Workload and workload intensity	Yes	22	5.4%	195	47.7%	44	10.8%
	No	18	4.4%	117	28.6%	13	3.2%
Emotional demands	Yes	6	1.5%	20	4.9%	2	0.5%
	No	34	8.3%	292	71.4%	55	13.4%
Expectations and feedback	Yes	37	9.0%	304	74.3%	57	13.9%
	No	3	0.7%	8	2.0%	0	0.0%
Organizational culture	Yes	30	7.3%	274	67.0%	55	13.4%
	No	10	2.4%	38	9.3%	2	0.5%
Supervisor support	Yes	30	7.3%	272	66.5%	56	13.7%
	No	10	2.4%	40	9.8%	1	0.2%

Overall, the findings indicate marital status, workload, role clarity, constructive organizational feedback, organizational culture, and supervisor support as significant factors influencing the job performance of the employees. The study reveals that education, experience, work pressure, feedback systems, organizational culture, and supervisor support are key factors in measuring stress levels in individuals. Therefore, 51.2% of the participants (n =169) show satisfactory performance alongside moderate stress levels and are highly sensitive to expectations and feedback, organizational culture, and supervisor support.

## Discussion

The job performance of health workers is a multifaceted concept that integrates various aspects of delivering high-quality care. However, health workers face numerous challenges in providing optimal care, including limited resources and infrastructure, high patient-to-nurse ratios, shortages of specialized staff, limited opportunities for professional development and training, and other factors that contribute to frustration, anxiety, and, ultimately, stress [26].

A previous study revealed significant occupational stress levels among healthcare professionals, with 59.6% identifying emotional interactions with patients' relatives as a frequent stressor (16.9%, always; 20.6%, usually; and 22.1%, often). High workload and long working hours were also prominent, reported by 55.8% of the participants as a frequent cause of stress (14.7%, always; 22.4%, usually; and 18.7%, often). Stress caused by quick decision-making and mobbing was noted by 22.1% and 28.5% of the respondents, respectively. Statistically significant associations were observed between stress frequency and physical and emotional health: 60% of healthcare professionals experiencing very frequent stress reported severe disruptions to social activities (p ≤ 0.001), and 71.4% noted that stress interfered substantially with work performance (p ≤ 0.001). These findings emphasize the critical need for stress management interventions, with healthcare professionals ranking communication (mean score = 4.55), safe work environments (mean score = 4.52), and eliminating mobbing (mean score = 4.46) as the most effective strategies [3].

The effect of occupational stress on job performance among health workers in two Bahraini governmental hospitals was investigated. The findings indicate a concerning trend where a large proportion of the workforce experiences moderate stress (76.3%) while a significant minority reports high stress (13.9%). This suggests a high prevalence of occupational stress among health workers in these hospitals. These findings are consistent with those from previous studies. For instance, a study by Hasan and Mahdi shows that 86.5% of physicians and nurses in governmental and private hospitals regularly experience chronic occupational stress [19].

Based on the results of this study, the participants with a bachelor's degree and more than 11 years of experience were more vulnerable to occupational stress, consistent with previous study findings in Bahrain [18,19]. Furthermore, occupational stress was significantly affected by workload and workload intensity, emotional demands, expectations and feedback, organizational culture, and supervisor support [3].

Regarding job performance, most health workers in this study perceived their job performance as satisfactory (63.1%), which aligns with findings by Al Ubaidi et al. (2020), where job satisfaction among primary care physicians in Bahrain was found to be approximately 60.5% despite the challenges of burnout and stress [27]. This study also identified that workload intensity, role clarity, feedback, organizational culture, and supervisor support significantly impact job performance, consistent with the findings of Zhang et al. (2024), which highlighted that a positive psychosocial work environment, including interpersonal relations and leadership, is a key determinant of well-being and job effectiveness [28].

Regarding the relationship between occupational stress and job performance, this study observed a strong negative correlation. As stress levels increased, job performance significantly declined. This is consistent with findings by Abuzeyad et al. (2021), who reported that higher levels of stress and burnout in emergency physicians in Bahrain correlated with reduced job satisfaction and performance [29]. Similarly, Malik et al. (2017) found that high levels of stress among Bahraini primary care physicians were linked to dissatisfaction and lower productivity​ [18]. These results collectively underscore the critical need for interventions to mitigate occupational stress and enhance job performance among healthcare professionals.

These findings emphasize the significant role of workplace factors, such as workload, expectations and feedback, organizational culture, and supervisor support, in managing stress and improving job performance. Several strategies have been shown to effectively manage occupational stress and enhance healthcare workers' performance. According to the Cochrane Review (2006) on occupational stress management programs, interventions such as employee assistance programs, supervisor training, and promoting work-life balance can significantly reduce stress levels and improve workplace outcomes [20]. Furthermore, studies highlight that fostering a positive organizational culture, particularly one that encourages open communication, collaboration, and psychological safety, helps mitigate stress and improve employee performance [30]. Establishing clear communication structures, redesigning work to minimize bureaucratic burdens, and maintaining optimal staffing ratios are essential strategies to address workplace stress and enhance job performance​​ [20,30].

Strengths and weaknesses

This study has several strengths that enhance its validity and contribution to the field. The use of validated tools, such as the Perceived Stress Scale (PSS) and the Individual Work Performance Questionnaire (IWPQ), ensures reliable and consistent measurement of key variables. The inclusion of healthcare workers from two governmental hospitals in Bahrain provides a diverse sample, offering valuable insights into workplace factors affecting occupational stress and job performance. Moreover, the study addresses critical workplace issues, such as organizational culture, workload, and supervisory support, while providing actionable recommendations for mitigating stress. However, there are limitations to consider. The cross-sectional design restricts the ability to establish causation, as only associations between stress and performance could be assessed. The reliance on self-reported data introduces potential bias, as the participants may over- or underreport their experiences. Additionally, the generalizability of the findings is limited, as the study focuses solely on two hospitals and excludes incomplete responses, which may have impacted the representation of the broader healthcare workforce.

Limitations

The data were collected using a self-developed questionnaire; therefore, the results may contain inaccuracies due to possible misunderstandings of some questions and respondent dishonesty. Moreover, the cross-sectional design of the study restricts its ability to provide insights beyond the specific point in time of data collection. Furthermore, the study was conducted in only two governmental hospitals, which may limit the generalizability of the findings.

Recommendations

This research highlights several workplace factors that significantly influence occupational stress and job performance. Addressing these factors is essential to implementing effective measures for managing their effects. For instance, organizations should focus on developing targeted interventions such as resilience training, leadership development programs, and policies that promote work-life balance. However, while this research focuses on commonly discussed factors in the literature, other factors warrant further investigation in future studies. Future research should explore the impact of emerging workplace dynamics, such as redesigned workflows, the integration of digital technologies, and cultural diversity, on occupational stress and performance. Additionally, longitudinal studies are needed to examine the long-term effects of stress management interventions and to identify causal relationships between workplace factors and employee outcomes. Investigating the role of personal resilience, adaptive coping mechanisms, and team-based support systems could also provide deeper insights into effective strategies for reducing stress and improving job performance. These future directions will help build a comprehensive understanding and inform more robust, evidence-based organizational practices.

## Conclusions

This study highlights moderate to high levels of occupational stress among healthcare workers in two governmental hospitals in Bahrain, with most participants rating their job performance as satisfactory. Occupational stress negatively affects job performance, influenced by factors such as workload, feedback, organizational culture, and supervisory support. Stress levels vary based on years of experience, education, and emotional demands, while job performance is shaped by marital status and role clarity. To mitigate these issues, healthcare organizations should implement stress management training, promote equitable workloads, and foster a supportive organizational culture.

Future research should explore the long-term effects of occupational stress and the effectiveness of management interventions through longitudinal studies. Investigating emerging factors, such as digital tools, team dynamics, and cultural diversity, could provide deeper insights into reducing stress. Addressing these areas will support the development of evidence-based strategies for healthier and more productive healthcare workplaces.

## Appendices

Tables 9-11 show occupational demographics, the Perceived Stress Scale, and the Individual Work Performance Questionnaire.

**Table 9:** Demographics and workplace

Demographic information	
1	Age
2	Sex: 1) male / 2) female
3	Professional status: 1) medicine / 2) dentist / 3) nurse / 4) others
4	Marital status: 1) single / 2) marriage / 3) others
5	Hospital: 1) Salmaniya Medical Complex / 2) Psychiatric Hospital
6	Educational level: 1) bachelor's / 2) master's / 3) occupational certified / 4) others
7	Years of work experience
Workplace factors	
8	Do you feel overwhelmed by your workload most days? 1) yes / 2) no
9	Do you have control over your schedule and breaks? 1) yes / 2) no
10	Is your role clearly defined and understood by colleagues and supervisors? 1) yes / 2) no
11	Do you feel supported by your colleagues and supervisor during challenging times? 1) yes / 2) no
12	Do you feel emotionally drained after interacting with difficult patients or families? 1) yes / 2) no
13	Do you experience physical discomfort or pain due to the demands of your work? 1) yes / 2) no
14	Do you feel like your achievements are recognized and valued by colleagues and supervisors? 1) yes / 2) no
15	Do you feel like your achievements are recognized and valued by colleagues and supervisors? 1) yes / 2) no
16	Do you feel like you are able to maintain a healthy balance between work and personal life? 1) yes / 2) no
17	Are employees encouraged to speak up and share concerns or suggestions for improvement? 1) yes / 2) no

**Table 10:** Perceived Stress Scale

For each question, choose from the following alternatives: 0, never; 1, almost never; 2, sometimes; 3, fairly often; 4, very often	
18	In the last month, how often have you been upset because of something that happened unexpectedly? ________
19	In the last month, how often have you felt that you were unable to control the important things in your life? ________
20	In the last month, how often have you felt nervous and stressed? ________
21	In the last month, how often have you felt confident about your ability to handle your personal problems? ________
22	In the last month, how often have you felt that things were going your way? ________
23	In the last month, how often have you found that you could not cope with all the things that you had to do? ________
24	In the last month, how often have you been able to control irritations in your life? ________
25	In the last month, how often have you felt that you were on top of things? ________
26	In the last month, how often have you been angered because of things that happened that were outside of your control? ________
27	In the last month, how often have you felt that difficulties were piling up so high that you could not overcome them? ________

**Table 11:** Individual Work Performance Questionnaire (IWPQ)

For each question, choose from the following alternatives: 0, seldom; 1, sometimes; 2, regularly; 3, often; 4, always	
Task performance (TP) scale	
28	TP1: I was able to plan my work so that I finished it on time.
29	TP2: I kept in mind the work result I needed to achieve.
30	TP3: I was able to distinguish main issues from side issues.
31	TP4: I was able to carry out my work well with minimal time and effort.
32	TP5: I planned my work optimally.
Contextual performance (CP) scale	
33	CP6: On my initiative, I started new tasks when my old tasks were completed.
34	CP7: I took on challenging tasks when these were available.
35	CP8: I worked on keeping my job-related knowledge up to date.
36	CP9: I worked on keeping my work skills up to date.
37	CP10: I came up with creative solutions for new problems.
38	CP11: I took on extra responsibilities.
39	CP12: I continually sought new challenges in my work.
40	CP13: I actively participated in meetings and/or consultations.
Counterproductive work behavior (CWB) scale	
41	CWB14: I complained about unimportant issues at work.
42	CWB15: I made problems at work bigger than they were.
43	CWB16: I focused on the negative aspects of a situation at work instead of the positive aspects.
44	CWB17: I talked to colleagues about the negative aspects of my work.
45	CWB18: I talked to people outside of the organization about the negative aspects of my work.
